# Effects of Bioactive Glasses (BGs) on Exosome Production and Secretion: A Critical Review

**DOI:** 10.3390/ma16114194

**Published:** 2023-06-05

**Authors:** Sara Gorgani, Seyede Atefe Hosseini, Andrew Z. Wang, Francesco Baino, Saeid Kargozar

**Affiliations:** 1 Tissue Engineering Research Group (TERG), Department of Anatomy and Cell Biology, School of Medicine, Mashhad University of Medical Sciences, Mashhad 917794-8564, Iran; sara.gorgani70n@gmail.com; 2 Department of Medical Biotechnology and Nanotechnology, Faculty of Medicine, Mashhad University of Medical Sciences, Mashhad 917794-8564, Iran; atefehosseini20@gmail.com; 3 Department of Radiation Oncology, Simmons Comprehensive Cancer Center, UT Southwestern Medical Center, 5323 Harry Hines Blvd, Dallas, TX 75390, USA; andrew.wang2@utsouthwestern.edu; 4 Institute of Materials Physics and Engineering, Department of Applied Science and Technology, Politecnico di Torino, Corso Duca degli Abruzzi 24, 10129 Torino, Italy

**Keywords:** bioactive glasses (BGs), ion release, drug delivery, exosomes, tissue engineering, wound healing

## Abstract

There is an increasing trend toward the application of bioactive glasses in different areas of biomedicine, including tissue engineering and oncology. The reason for this increase is mostly attributed to the inherent properties of BGs, such as excellent biocompatibility, and the ease of tailoring their properties by changing, for example, the chemical composition. Previous experiments have demonstrated that the interactions between BGs and their ionic dissolution products, and mammalian cells, can affect and change cellular behaviors, and thereby govern the performance of living tissues. However, limited research exists on their critical role in the production and secretion of extracellular vesicles (EVs) such as exosomes. Exosomes are nanosized membrane vesicles that carry various therapeutic cargoes such as DNA, RNA, proteins, and lipids, and thereby can govern cell–cell communication and subsequent tissue responses. The use of exosomes is currently considered a cell-free approach in tissue engineering strategies, due to their positive roles in accelerating wound healing. On the other hand, exosomes are known as key players in cancer biology (e.g., progression and metastasis), due to their capability to carry bioactive molecules between tumor cells and normal cells. Recent studies have demonstrated that the biological performance of BGs, including their proangiogenic activity, is accomplished with the help of exosomes. Indeed, therapeutic cargos (e.g., proteins) produced in BG-treated cells are transferred by a specific subset of exosomes toward target cells and tissues, and lead to a biological phenomenon. On the other hand, BGs are suitable delivery vehicles that can be utilized for the targeted delivery of exosomes to cells and tissues of interest. Therefore, it seems necessary to have a deeper understanding of the potential effects of BGs in the production of exosomes in cells that are involved in tissue repair and regeneration (mostly mesenchymal stem cells), as well as in those that play roles in cancer progression (e.g., cancer stem cells). This review aims to present an updated report on this critical issue, to provide a roadmap for future research in the fields of tissue engineering and regenerative medicine.

## 1. Introduction

Exosomes are nanosized biological structures that are secreted by different types of mammalian cells into physiological fluids in the body. They are generally categorized as a subfamily of extracellular vesicles (EVs). Although exosomes were first considered as the “waste bags” of living cells, experimental studies have revealed their critical roles in the cell signaling pathways of the body [1]. They are involved in local (autocrine and paracrine) and distant (endocrine) signaling via transferring their contents. Nowadays, it is well-understood that every single alteration in tissue condition (e.g., cell proliferation, apoptosis, angiogenesis) is associated with cell–cell or cell–extracellular matrix (ECM) communications. Thus, exosomes have been found to occupy a unique place in governing tissue performance in the body. In fact, exosomes can carry biomolecular cargoes (e.g., proteins, nucleic acids, lipids) between different cells and tissues. It is stated that almost all mammalian cells, both normal and cancerous, may secrete their specific exosomes with a determined biochemical component. For example, exosomes have been proven to play a substantial role in cancer progression, survival, and metastasis [2,3]. Therefore, they can be considered and tracked as biomarkers for different healthy and unhealthy tissues [4]. As they have the ability to establish biochemical communication, biosafe exosomes are utilized as suitable delivery vehicles for various biomolecule and drug cargoes in medicine. While several clinical trials have demonstrated the satisfactory therapeutic capacity of exosomes in wound healing, their limited production, and the problems that remain regarding their isolation, still limit their wide-spread use in clinics [5]. Therefore, the stimulation of cells by the activation of special proteins and signaling pathways has been proposed in order to achieve higher yields of exosomes.

With their exceptional regenerating potential, bioactive glasses (BGs) have gained a special place in tissue-engineering approaches [6,7]. These synthetic biocompatible materials were first utilized for managing hard-tissue diseases and disorders; however, they have been found to be beneficial for managing soft tissues as well [8,9,10,11]. The reason for this is attributed to the great compositional versatility of BGs, which can be properly designed to achieve various physico-chemical, mechanical, and biological characteristics [12]. In addition to their chemical composition, other aspects of BGs, including morphology and surface characteristics, may affect cellular behaviors, and thereby potentially promote tissue repair and regeneration. Despite all the aforementioned advantages of BGs in biomedicine, some issues should be considered when biocompatible glasses are used for the treatment of human diseases and disorders. These issues include the sharp increase in local pH, possible adverse effects on cells due to ion release, and the low mechanical strength and fracture-toughness of BGs and BG-based constructs [13]. However, the release of therapeutic ions from BG particles is recognized as the main factor in determining their biological performance [14,15]. Most reported studies have shown the possible effects of glass dissolution products on cell metabolism, growth, and proliferation, as well as gene and protein expression. Still, studies addressing the impact of BGs on the production and secretion of exosomes, either in normal cells or cancer cells, have been limited [16]. The few available experimental studies on this topic suggest that BGs can induce the secretion of exosomes in mesenchymal stem cells (MSCs) through the nSMases and the Rab family of small GTPases, which play a critical role in stimulating endothelial cells (ECs) to overexpress genes involved in angiogenesis (new-blood-vessel formation) [17]. To gather the information presented in this review, we applied the keywords of “exosome”, “extracellular vesicles”, “bioactive glass”, “bioglass”, “tissue engineering”, and “cancer research” to Google Scholar, Pub Med, Scopus, and Web of Science. To the best of our knowledge, this is the first article on the potential effects of BGs on the production and release of exosomes from mammalian cells, and their beneficial impact on tissue engineering and regenerative medicine.

## 2. Bioactive Glasses (BGs): A Unique Class of Materials in Medicine

Bioactive glasses (BGs) are categorized as the third generation of biomaterials that benefit from highly reactive surfaces. These biocompatible substances can trigger special biological activities once implanted into the body [18]. The first commercialized BG (45S5 Bioglass^®^) was developed by Larry Hench in 1969 at Florida University, with the aim of treating defective hard tissues [19]. The 45S5 Bioglass^®^ is known as the parent of silicate BGs, with the chemical composition of 46.1% SiO_2_, 24.4% Na_2_O, 26.9 CaO, and 2.6% P_2_O_5_ (mol %) [12]. Over time, two other categories of BGs were developed, phosphate and borate, which exhibit different properties compared to silicate glasses. Utilizing various synthesis methods, mostly the melt-quench and sol–gel routes, it is feasible to produce BGs in different sizes and shapes (e.g., nanostructured powder, fibers), with tunable physico-chemical, mechanical, and biological properties. Compared to dense melt-quenched BG particles, sol–gel-derived BGs display a porous structure that makes them suitable substances for drug-delivery applications. Up to now, a broad range of natural and synthetic therapeutic substances have been loaded into, and delivered from, BGs, to desired sites in the body, with the aim of performing targeted treatments, such as cancer therapy [20]. BGs can be easily manufactured into 3D scaffolds with different architectures, for implantation into damaged tissues. Furthermore, BG particles, mostly nanosized particles, can be added to polymeric substrates to develop composite constructs (e.g., electrospun nanofibrous composites), also in the form of scaffolds with improved properties (e.g., biological performance) [21]. Currently, BG products with different compositions and shapes are available on the market for treating a wide range of tissue damages and injuries [22,23,24,25]. The capability of BGs to form rapid, powerful, and long-lasting bonds with living body tissues has led to their extensive application in tissue engineering and regenerative medicine [26,27]. It should be highlighted that the use of BGs in medicine is not limited to tissue-engineering strategies; specific formulations of BGs have been tested in cancer treatment as well. The anticancer effects of BGs can be improved by adding certain elements to their composition. For instance, tellurium-, magnesium-, and silver-doped BG nanoparticles have previously been synthesized and successfully tested against tumor cells [28,29,30]. There are several studies in the literature that indicate the usability of BGs in cancer hyperthermia, photothermal therapy, and anticancer drug delivery [31,32]. Anticancer BG compositions show promise in being more versatile, and even safer, materials for cancer therapy, compared with commonly used inorganic particles (e.g., iron oxide nanoparticles). However, no clinical trials have yet been conducted to investigate the anticancer potential of BGs in vivo.

Several experimental studies have shown that the therapeutic effects of BGs mainly depend on ion release from their structure into the physiological environment. Accordingly, a wide range of elements have been added to the basic composition of BGs to render specific biological features (e.g., promoted angiogenesis). For example, the release of cobalt ions (Co^2+^) from the glass network into biological environments can lead to the overexpression of a series of genes involved in hypoxia (e.g., hypoxia-inducible factor 1alpha (HIF-1α)), that can in turn improve angiogenesis, via enhancing extracellular matrix (ECM) degradation, as well as stalk-cell proliferation and migration [33]. Molecular biology techniques have allowed glass researchers and scientists to gain a deeper understanding of how BGs affect cell behaviors and tissue performance. In this regard, the influence of ionic dissolution products from BGs on the biosynthesis of exosomes may indeed be one of the most interesting topics in the field, since it can shed light upon the regenerative capacity of these biomaterials.

## 3. Exosomes: Multifunctional Nanovectors for Biodelivery

**Figure 1:**
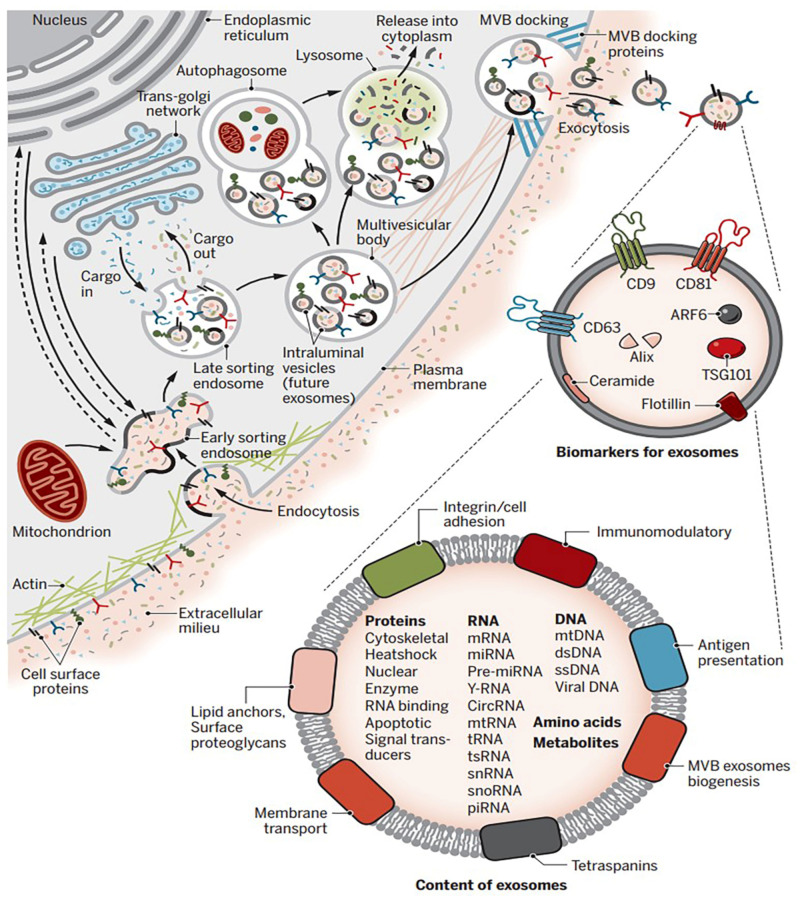
The schematic illustration of exosome biogenesis. In summary, the exosome formation starts with an intra-luminal invagination/endocytosis of the plasma membrane. The primary-formed vesicle is called an early sorting endosome (ESE), which then matures to a late sorting endosome (LSE). The multivesicular body (MVB), which contains future exosomes, is created because of secondary invaginations in the LSE. The MVB can undergo degradation by fusing to a lysosome, or releasing the exosomes to the extracellular space. Released exosomes have distinct compositions of surface markers, and may contain various types of bio-substances. Reproduced with permission from [58].

Exosomes represent biologic membrane bilayers, which are made and secreted into physiological fluids by all cell types (normal and tumor cells). While “exosome” and “extracellular vesicle (EV)” are often used interchangeably in the literature, it should be emphasized that EV is a general term used for micro-vesicles with different release mechanisms and sizes (diameter 100 to 1000 nm). Contrastingly, exosomes, as the smallest family of EVs, have a diameter between 40 and 150 nm [34]. In 1967, Wolf detected these structures, and described them as “platelet dusts”. Twenty years later, Rose Johnstone first named these bilayer membranes as exosomes [2,35]. Their simple structure is composed of biological molecules such as DNA, RNA (mRNA, tRNA, lncRNA, miRNA, and mtDNA) [36], proteins, lipids, cytokines, transcription factor receptors, and any other bioactive molecule that is present in the secreting cell [37]. The exact mechanism of exosome formation is still under discussion, but the classic formation of exosomes begins with an invagination of the plasma membrane, and continues with the accumulation of bioactive substances in the primary-formed vesicles, named “early stage endosomes” or ESEs. ESEs are modified into endocytosis-sorting complexes, and form “late sorting endosomes” or LSEs, which in turn became “multivesicular bodies” or MVBs. The MVBs integrate with the plasma membrane, and eventually release the final products, exosomes (Figure 1) [4]. It should be mentioned that exosome formation can also occur directly, when the contents bud directly and immediately from the plasma membrane [38]. The best-understood pathway of exosome formation is called endosomal-sorting-complexes-required-for-transport-dependent (ESCRT-dependent). Its subcomplexes are the main cargo-sorting complexes in the cytoplasm, and they recognize the inwarded endosomes, and then guide them toward further modifications, in order to form MVBs and release them as exosomes. Other ESCRT-independent pathways include the ceramide and tetraspanin family-mediated pathways [39].

As mentioned above, mammalian cells can secrete different exosomes with distinct compounds (tissue-specific) as a representative of the cell-source phenotype. These small structures can facilitate paracrine communication between cells and, therefore, determine a different fate for recipient cells [34,40]. Exosomes are present in nearly all biological fluids (e.g., urine, breast milk, saliva, plasma), and their biogenesis and secretion can be induced or regulated in physiologic and pathologic conditions by proteins, by various signaling pathways, and also by engineered bioactive materials [4]. As previously mentioned, exosomes can be secreted by normal cells (e.g., mesenchymal stem cells (MSC), NK cells, T cells, B cells, macrophages, dendritic cells (DC)), as well as different tumor cell lines (e.g., metastatic colorectal cancer cells) [41]. Depending on their nature and origin, exosomes can proceed different biological processes by facilitating intercellular communication. MSC-derived exosomes are one of the most studied groups of exosomes with confirmed positive wound-healing impacts (e.g., skin scars, diabetes-induced cardiovascular injury) [42,43,44,45,46]. In addition to preclinical studies, there are several clinical trials in which exosomes have been successfully applied in the treatment of different ulcers (e.g., skin-related ulcers, diabetic foot ulcers, cesarean-section scars) [5,47,48]. Therefore, the use of exosomes, regardless of their source, has become very popular thanks to their therapeutic effects (e.g., anti-inflammatory and proangiogenic activities). Moreover, the low immune-system stimulation and nano-size, which helps them to pass through biological barriers, have established a novel branch of therapies called “cell-free therapy” [16]. In this approach, isolated exosomes can be administered as therapeutic cargoes via different routes (e.g., injection, inhalation). It should be noted that these isolated exosomes can contain natural biomolecules without any external manipulations, or carry certain types of biomolecules (e.g., mRNS, miRNA, drugs) which are added artificially using different methods (e.g., the cellular-nanoporation method), in order to induce a special biological process or deliver a drug to targeted cells [49]. In spite of all the positive aspects mentioned, the isolation and purification of exosomes are still recognized as a main unsolved challenge for medical applications. One reason is associated with the extensive variation in the size, content, and source of exosomes. Additionally, the similarity of exosomes to other biphasic biogenic structures (e.g., lipoproteins) make it even more difficult to precisely perform their purification. 

Various methods are used for exosome isolation, including ultracentrifugation (UC) techniques [50], size-based isolation [51], immunoaffinity chromatography [52], and polymer precipitation [53]. UC is the most commonly used, and also the gold-standard method for isolation. With UC, exosomes are separated from other cell components according to size and density differences. This method comprises two steps: (I) removing cells and other large-sized exosomes through operating several low–medium-speed centrifuges, and (II) isolating exosomes at high-speed centrifugal force, and washing them with PBS to eliminate impurities (e.g., contaminating proteins) [54]. Different characterization methods should be performed in order to verify whether the isolated substances are actually exosomes or not. Morphology and size evaluations can be carried out by utilizing a transmission electron microscope (TEM), nanoparticle tracking analysis technology (NTA), and dynamic light scattering (DLS). Moreover, Western blotting and flow cytometry can be performed to identify exosome marker protein (e.g., membrane protein or lipid rafts) [55,56]. Another challenge is related to the limited capacity of cells to secrete exosomes. The cell type, viability, and surrounding microenvironment can affect the yield of exosome production. Therefore, obtaining large doses of exosomes for clinical use has remained a major challenge [57]. One proposed solution is to use specific biomaterials and scaffolds to stimulate cells toward a higher production of exosomes. In this regard, it has been previously shown that BG-based scaffolds can activate exosome production in mammalian cells by releasing specific ions into cell-culture media [17].

## 4. Impact of BGs on Exosome Biogenesis

It is now well-acknowledged that multiple types of biomaterials can accelerate tissue repair and regeneration, through stimulating various cells (e.g., MSCs) to secret specific kinds of exosomes. Exosome formation in cells can be stimulated by bioactive materials, through the activation of certain signaling pathways (autocrine, paracrine, and endocrine) that cause alterations in the behaviors of target cells. The composition of the applied biomaterials directly affects the type and activity of the released exosomes [59]. Previous studies have confirmed that bioceramics can influence the production of specific types of exosomes in mammalian cells, which can lead to an improved tissue-healing process through the activation and enhancement of biological phenomena such as osteogenesis and angiogenesis [60,61,62,63,64]. In this regard, Lin et al. showed that strontium-substituted calcium silicates (Sr-CS) can promote neovascularization through the activation of human umbilical vein endothelial cells (HUVECs) to produce and secrete proangiogenic miR-146a-containing exosomes, and the inhibition of Smad4 and NF2 proteins [65].

The same scenario was reported regarding BGs. Recent experimental studies have clarified that BGs can impact the wound-healing process through the stimulation of cells to produce and secrete certain types of exosomes. This has provided a wider and clearer picture of the therapeutic effects of BGs in accelerating the wound-healing process. 

**Figure 2:**
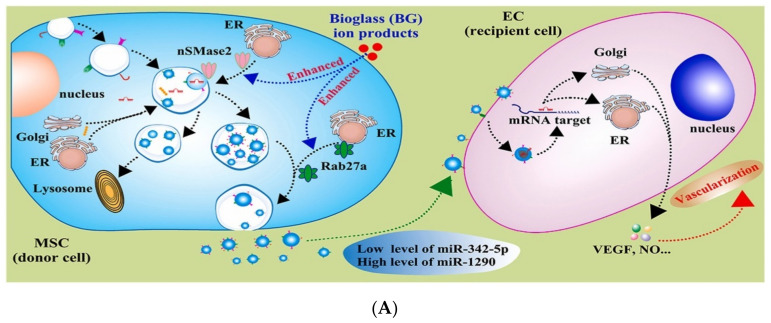

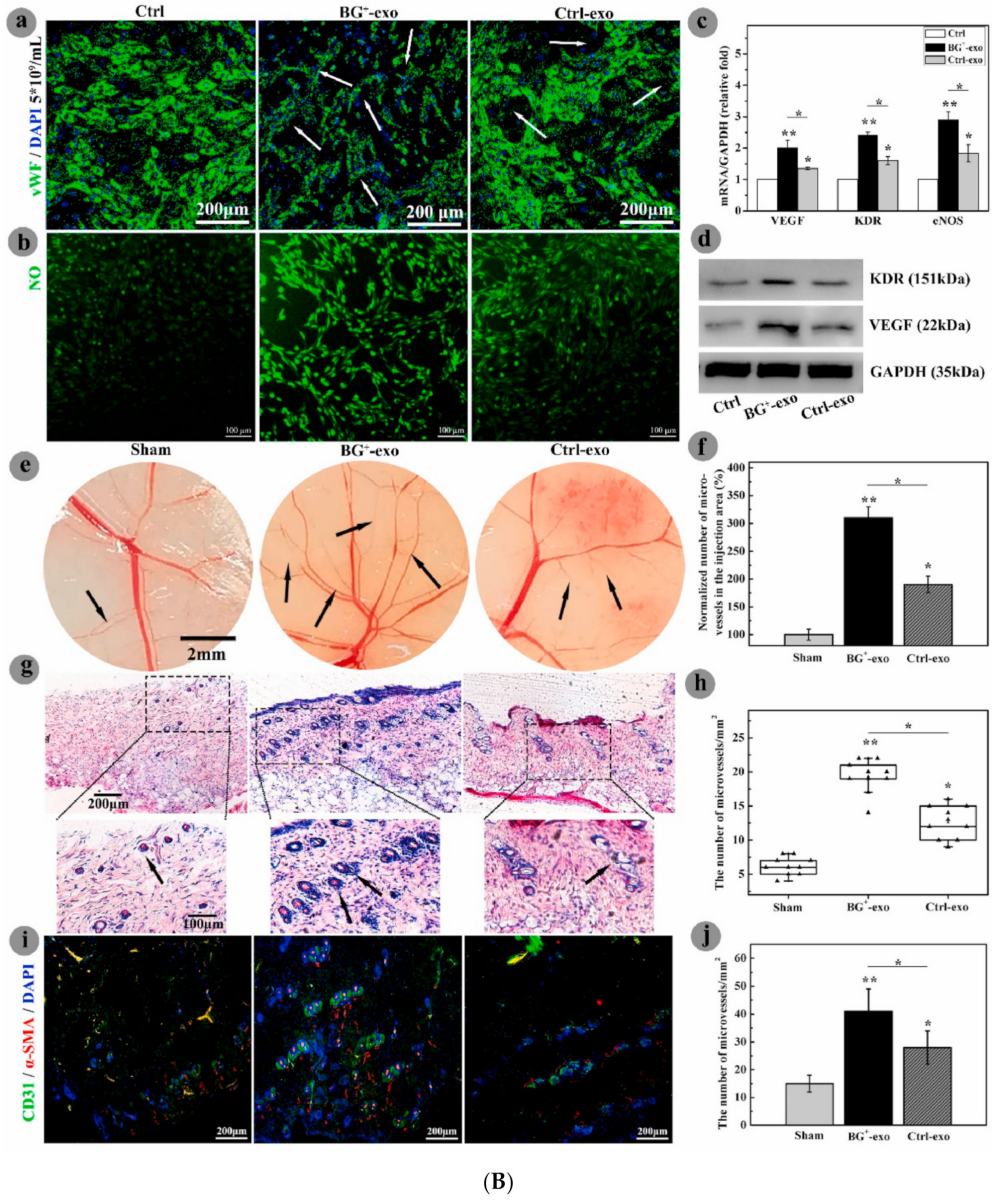
(**A**) A schematic representation of the potential effects of BGs on exosome production. As depicted, the ionic dissolution products released from BGs can stimulate the production and secretion of specific exosomes in MSCs, which can affect the behavior of endothelial cells (ECs) through defined signaling pathways in favor of promoted angiogenesis. (**B**) The effect of BGs in the enhancement of exosome production from MSCs, and the consequent promoted vascularization in vitro and in vivo: (**a**) vWF/DAPI staining, (**b**) NO staining, (**c**) expression of VEGF, KDR, and eNOS genes, (**d**) tracking of KDR and VEGF proteins in ECs co-cultured with MSC-derived exosomes in vitro for two days, (**e**,**f**) light-field images, and the quantitative analysis on observable micro-vessels, (**g**,**h**) H&E-stained skin samples at the injection area, and quantitative analysis of micro-vessel-like structures, and (**i**,**j**) the double-immunofluorescence images of skin samples stained with α-SMA and CD31 at the injection area, and quantitative analysis of the tagged micro-vessels. * *p* < 0.05 and ** *p* < 0.01 show the significant difference between the treatment groups and the corresponding sham/control group. (White and black arrows show capillary-like networks and blood vessels, “BG^+^” means BG stimulated MSC exosomes, and triangles indicate individual data points). Reproduced under CC license from [17].

The family of neural sphingomyelinases (nSMases), and the Rab family of small GTPases, are groups of well-identified proteins that are involved in the ESCRT-independent biosynthesis of exosomes from cells [66,67]. It has been reported that BG-dissolution products can affect these proteins in MSCs, in favor of tissue repair and regeneration. For example, Lu et al. showed that the ionic dissolution products of 45S5 Bioglass^®^ can enhance the exosome release of MSCs. In fact, they found that the overexpression of nSMases and the Rab27 family in treated cells can enhance the nSMase and Rab GTPase pathways, respectively. Interestingly, the contents of exosomes released from the BG-treated MSCs were different from those that were secreted by the cells receiving no external stimulation. The treated MSCs secreted exosomes with a decreased level of microRNA-342-5p, and increased amounts of microRNA-1290 at the same time. These exosomes were confirmed to be internalized by endothelial cells (ECs), and led to the overexpression of angiogenesis-related genes, including vascular endothelial growth factor (VEGF), VEGF receptor 2 (KDR), and endothelial nitric oxide synthase (eNOS). Indeed, the in vivo administration of MSC-derived exosomes resulted in the generation of a significant number of micro-vessels in the injection region, compared with control groups (see Figure 2) [17].

**Figure 3:**
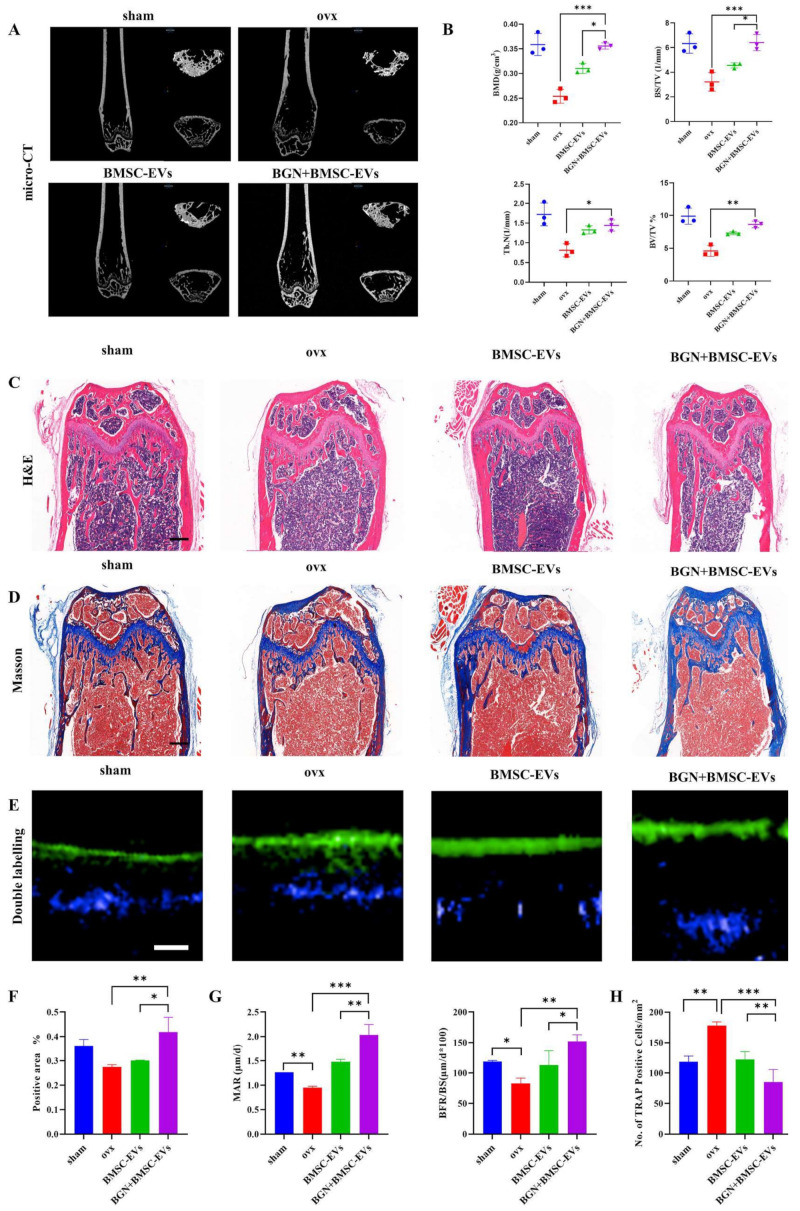
The inhibitory impact of BGN + BMSC-Evs on bone resorption in vivo: (**A**) micro-CT image of the femur, (**B**) Histomorphometry analysis of BMD, Tb. N, BS/TV, and BV/TV (symbols show scatter dot points) (**C**) H&E-staining images (scale bar, 500 μm), (**D**) Masson staining images (scale bar, 500 μm), (**E**) image of newly formed bones indicated by double labeling of tetracycline and calcein (scale bar, 20 μm), (**F**) volume fraction indicated from the collagen fiber analysis of the sham femurs, OVX, BGN + BMSC-EVs-treated OVX, and BMSC-EVs-treated OVX groups, (**G**) bone histomorphometric analysis of the MAR and BFR/BS in the femur of the treatment groups, and (**H**) histomorphometric analysis for counting osteoclasts in the samples. * *p* < 0.05, ** *p* < 0.01 and *** *p* < 0.001 demonstrate the significant difference between the treatment groups and the corresponding sham/control group. Reproduced with permission from [16].

Lithium-doped glass-ceramic (Li-BGC) scaffolds were prepared based on 45S5 BG, and their roles in the mediation of cell–cell communications between bone marrow stromal cells (BMSCs) and ECs were explored. The obtained results demonstrated that the release of Li^+^ ions from BGCs can enhance neovascularization in vitro and in vivo. To elaborate: the Li^+^ ions stimulated the secretion of specific BMSC-derived exosomes (BMSC-exo) containing proangiogenic miR-130a, which subsequently led to the downregulation of PTEN protein, and the activation of the AKT pathway. All these molecular phenomena resulted in elevated proliferation, migration, and tube formation of HUVECs in vitro. Moreover, the activation of the Wnt/β-catenin, AKT, and NF-κB signaling pathways was observed, and attributed to directly promoted new blood-vessel ingrowth in vivo [64]. Emphasizing the role of BGs in the treatment of bone-related diseases and disorders (e.g., osteoporosis), the nanosized BG particles (60SiO_2_-36CaO-4P_2_O_5_ mol %) were examined for their capabilities in stimulating stem cells to secrete exosomes in favor of inhibiting bone loss in osteoporotic animals. In this regard, Yang et al. developed a co-culture system in which mouse BMSCs were treated with BG nanoparticles (1 mg/mL). This experiment indicated that BGs could significantly inhibit osteoclast differentiation in RAW264.7 cells, suggesting their possible role in preventing osteoporosis. Subsequent in vivo experiments demonstrated that BG treatment can lead to inhibition of bone resorption (i.e., bone loss) in an osteoporosis mouse model via lncRNA NRON enrichment (Figure 3). Indeed, BG nanoparticles can induce the expression of the lncRNA NRON (a long non-coding RNA) in BMSCs, and their subsequent secretion through exosomes. The lncRNA NRON-containing exosomes can then be internalized by osteoclasts, which leads to the inhibition of the nuclear transfer of NFATc, thereby prohibiting the differentiation of osteoclasts [16].

**Figure 4:**
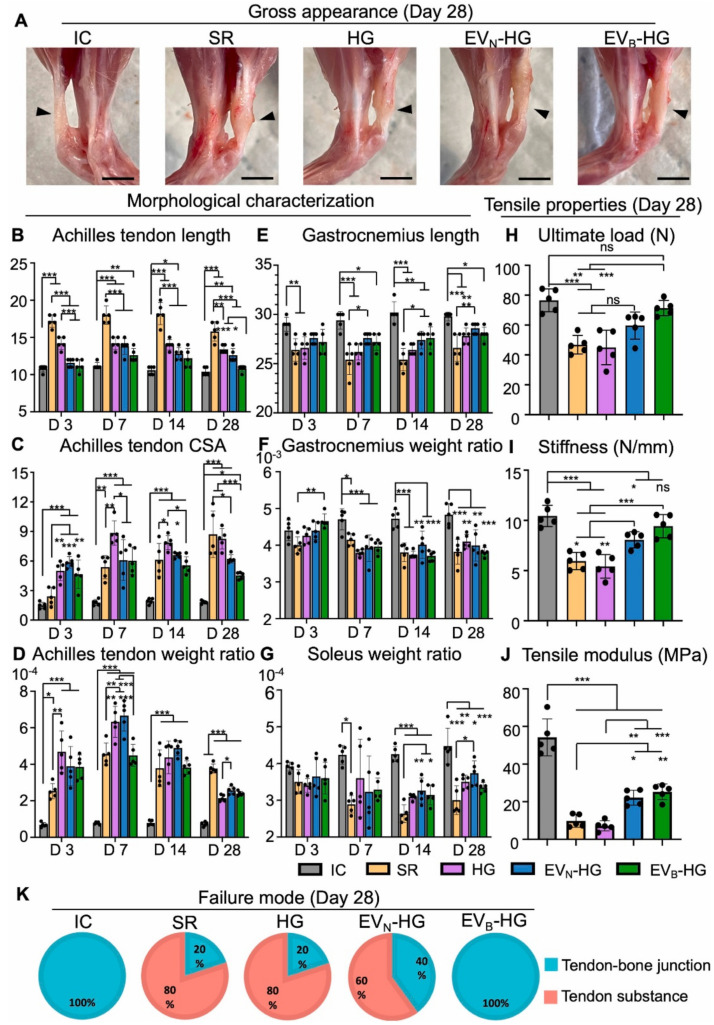
Morphological and biomechanical properties selectively enhanced in the calf-muscle–Achilles-tendon–calcaneus-bone complex by EV_B_-HG: (**A**) gross appearance of the Achilles tendon (showed by arrow) after 28 days (scale bar, 5 mm), (**B**) length of the Achilles tendon, (**C**) CSA of the Achilles tendon, (**D**) weight/body weight ratio of the Achilles tendon, (**E**) length of the Gastrocnemius muscle, (**F**) weight/body weight ratio of the Gastrocnemius muscle, (**G**) weight/body weight ratio of the soleus muscle, (**H**) ultimate load, (**I**) stiffness, (**J**) tensile modulus, and (**K**) failure mode. Data expressed as means ± SD; *n* = 5; * *p* < 0.05; ** *p* < 0.01; *** *p* < 0.001, and “ns” = not significant. (Black dots show scatter points). Reproduced with permission from [68].

In another recently published study, human-adipose-tissue-derived MSCs were treated with diluted eluates from the ionic dissolution of 45S5 Bioglass^®^ powders (particle size ∼38 μm), and EVs were then collected from treated cells (EV_B_) in order to investigate their effects in modulating macrophage polarization, angiogenesis, and tendon regeneration [68]. Compared to EVs isolated from untreated cells, the administration of the EV_B_ resulted in the up-regulation of a therapeutic miRNA profile (including miR-199b-3p and miR-125a-5p) that could promote angiogenesis through facilitating the specific ring spatial distribution of anti-inflammatory M2 Mφs macrophages and CD^31+^ ECs during tendon regeneration in vivo. The implantation of BG-elicited MSC-EV-laden GelMA hydrogels could improve tenogenesis, and reduce detrimental morphological changes, with no sign of heterotopic ossification in an acute rat Achilles-tendon-full-thickness rupture. From a biomechanical point of view, an enhanced ultimate load, stiffness, and tensile elastic modulus were observed in the tendon of animals receiving the EVB. The authors have concluded that this method can be considered a next-generation therapy for improving the functional reconstruction of damaged tendons (see Figure 4) [68].

## 5. Conclusions and Future Perspectives

An understanding of the language through which cells communicate is a basis for clarifying the mechanisms of different biological processes. Several molecular players have been identified that modulate cell–cell and cell–ECM signaling, and among them, exosomes represent a specific class of inter-cellular signaling objects that can affect biological phenomena such as tissue healing. In fact, the molecular cargoes of exosomes can trigger a cascade of signaling pathways in target cells, and lead to a biological response. Recent experimental evidence suggests that BGs can stimulate cells (e.g., MSCs) to synthesize and secrete exosomes with specific therapeutic cargoes that can have a positive effect on tissue repair and regeneration. In this regard, the proangiogenic capacity of silicate BGs have been linked with their potential in modulating the cargoes of MSC-derived exosomes (a decreased level of microRNA-342-5p, along with an increased microRNA-1290 level). However, among the few available published studies, most have been performed on a limited set of compositions of silicate-based glasses (e.g., 45S5 BG). Therefore, it is necessary to evaluate whether other subfamilies of BGs; i.e., phosphate and borate glasses, can affect the exosome constituent secreted by cells, and if so, to what extent. Since BGs have shown a variety of biological activities (e.g., anti-inflammatory, antioxidant), the possible role of BGs in the production of defined types of exosomes may provide better insight into their therapeutic potential in tissue engineering, regenerative medicine, and cancer research. Furthermore, the loading and delivery of exosomes using mesoporous BGs or 3D glass-based constructs (scaffolds) can be considered a novel cell-free approach for enhancing desired outcomes. 

In summary, BGs could perform a double function in the context of exosome applications for advanced therapy by: (i) acting as carriers for the release of previously secreted/produced exosomes, and (ii) producing a stimulatory effect on cells to produce exosomes through the release of appropriate ionic species. Of course, strategy (ii) potentially carries immeasurable advantages; for example: no longer requiring drugs, with their associated problems of high cost and side effects; and avoiding cells with problems relating to harvesting (if autologous cells), or immune rejection (if harvested from an external donor). Such an approach, although extremely fascinating, is nascent, and more studies are necessary to elucidate whether it is actually reproducible, safe, scalable from in vitro tests to animal studies and, eventually, to humans, and whether it can be relied on to achieve the desired action at a finely controllable level. Lastly, regulatory aspects, and the need for common protocols to be adopted in terms of the characterization and validation of its effects, will be key to the success of research in such a cross-disciplinary field.

## Data Availability

This paper is a review; therefore, data are available in the source publications listed in the bibliography.
